# In Vitro and In Vivo Evaluating Bioaccessibility, Bioavailability, and Antioxidant Activities of Butterfly Pea Flower Containing Bioactive Constitutes

**DOI:** 10.3390/foods13101485

**Published:** 2024-05-10

**Authors:** Fengyao Yu, Qinqin Yu, Ning Yin, Genlin Sun, You Peng, Yan Zeng, Yong Sun, Xiaoya Wang, Hua Zhang

**Affiliations:** 1 Department of Food Nutrition and Safety, College of Pharmacy, Jiangxi University of Chinese Medicine, Nanchang 330004, China; yufengyao@jxutcm.edu.cn (F.Y.); yuqinqin@jxutcm.edu.cn (Q.Y.); 202281704003@jxutcm.edu.cn (N.Y.); 20201004@jxutcm.edu.cn (G.S.); 15798033616@163.com (Y.Z.); 2 Jiangxi Province Engineering Research Center of Ecological Chemical Industry, Jiujiang University, Jiujiang 332005, China; trihydracid@126.com; 3 State Key Laboratory of Food Science and Technology, Nanchang University, Nanchang 330047, China; yongsun@ncu.edu.cn; 4 Chemical Biology Center, Lishui Institute of Agriculture and Forestry Sciences, Lishui 323000, China

**Keywords:** butterfly pea flower, anthocyanins, bioaccessibility, bioavailability, antioxidant

## Abstract

The antioxidant properties of butterfly pea flower (BF), which is rich in natural anthocyanins, have garnered significant attention. The impact of digestion and metabolism on BF extracts and evaluate their subsequent antioxidant activities in vivo were explored in the present study. After in vitro digestion, 42.03 ± 2.74% of total anthocyanins from BF extracts remained, indicating a negative influence of the digestion process on the bioaccessibility of phenolic compounds derived from BF. Furthermore, UPLC-LTQ-Orbitrap-MS^2^ analysis identified a total of four prototypes and twenty-seven metabolites in rat plasma or urine samples following the intake of BF extracts. The kinetics of key metabolites including delphinidin 3-glucoside (D3G), cyanidin-3-glucoside (C3G), and 4-hydroxybenzoic acid were subsequently determined in blood, and the Cmax values were 69.034 ± 8.05 nM and 51.65 ± 3.205 nM. These key metabolites derived from BF anthocyanins, including C3G and D3G, and flavonoid quercetin exhibited main antioxidant attributes that improved the plasmic and hepatic activities of various antioxidant enzymes and the total antioxidant capacity (T-AOC) and malondialdehyde (MDA) in a D-galactose-induced rat model. These findings provide insights into the bioaccessibility and bioavailability of bioactive constitutes derived from BF extracts, which are crucial for determining the actual efficacy of BF as well as developing functional foods based on BF.

## 1. Introduction

The butterfly pea (*Clitoria ternatea*) flower (BF), a member of the Fabaceae family, is widely cultivated in tropical and temperate zones, including India, Malaysia, Thailand, and the south of China. Fresh BFs have five petals shaped as butterflies with a distinguish bright blue color with a yellow or white center. Dried BFs are edible and conventionally consumed as flower tea or ingredients in blue rice [1]. In recent, BFs have been known for its high anthocyanin content, making it a promising natural food colorant resource [2,3]. Consequently, BF extracts received approval from the US Food and Drug Administration (FDA) in 2021 and are extensively utilized as natural colorants in the food and beverage industry. However, replacing synthetic food colors with natural alternatives has remained challenging due to their relatively high production costs. Nevertheless, advancements in biosynthesis technologies and industrialization over recent decades are expected to significantly improve the scalability and cost-efficiency of natural pigment production in the near future [4]. The antioxidant properties of these natural pigments have garnered significant attention from researchers and consumers alike [5]. As a result of increasing consumer demand for healthier foods, there has been an exponential growth in the market for these natural matrices as replacements for synthetic colorants.

However, blue pigments are a rarity in nature. The extracts from BF contain quadri-acylated delphinidin-based anthocyanins known as ternatins, which are stable natural blues [4]. The structural feature of ternatins relies on the acyl residue formed by hydroxycinnamic (HCA) moieties repeating and linking with sugar moieties, thereby enhancing the stability of anthocyanins present in BF under neutral and high pH conditions [2]. Consequently, these anthocyanins derived from BF extracts exhibit resistance to pH fluctuations, UV exposure, and enzymatic degradation. Apart from those, other polyphenols derived from BF extracts have been identified as derivatives of major anthocyanidins (delphinidin, cyanidin, and pelargonidin), as well as flavonols such as rutin and kaempferol derivatives. These bioactive constitutes are the main attributes for alleviating the low-grade inflammation in the host to lower the risk of developing metabolic syndrome (MetS) [6]. Hence, BF extracts may serve as optimal food resources for obtaining natural blue pigments that exert the antioxidant, anti-inflammatory, and anti-cancer actions [7]. In an animal model, the results of pharmacokinetic studies demonstrate that a quick rise in acylated anthocyanin levels in blood, indicating the stomach absorption being involved in the absorption of acylated anthocyanin [8]. Likewise, the absorption of the intact malvidin-3-*O*-(6-*O*-coumaroyl)-glucoside-5-*O*-glucoside from red wine was observed in human gastric cell lines, compared with its nonacylated counterpart [9]. Accordingly, the structural characteristics of acylated anthocyanins may exert a substantial influence on their bioavailability and subsequent bioactivity, particularly antioxidant activity. Those are necessary for further investigation in this study.

Importantly, even though anthocyanins known as natural antioxidants by scavenging reactive oxygen species (ROS) within the body, their radical scavenging activity was enhanced by acyl group [10]. The accumulation of ROSs causes the significant aggravation of antioxidant activity in vivo, leading to impaired redox balance and ultimately resulting in oxidative stress [11]. While the physiological level of ROS produced by the host is intended to eliminate invading pathogens and deplete malignant cells, excessive ROS can damage cellular molecules such as lipids, proteins, and DNA. The failure to clean up excessive ROS leads to progressive oxidative damage and eventual cell death [12]. The extracts of purple potatoes or carrots, also rich in acyl anthocyanins, exhibit potent scavenging ability to effectively neutralize excessive ROS [13]. These anthocyanins have been demonstrated to attenuate hydroperoxide-induced oxidative stress in intestinal epithelial cell lines by enhancing cellular antioxidant capacity, restoring antioxidant enzyme activities, and suppressing the production of pro-inflammatory cytokines [13]. A variety of anthocyanins derived from food resources play a chemical protective role through cell signal transduction and reduce oxidative damage. They are effective bioactive components that maintain the redox balance. Accordingly, the intake of anthocyanin-enriched foods can lower the risk of developing metabolic syndrome [14,15].

However, the bioaccessibility, bioavailability, and in vivo antioxidant properties of anthocyanins derived from BFs remain underexplored, limiting our understanding of their potential health benefits. Furthermore, comprehending the bioaccessibility, bioavailability, and related bioactivities of BF extracts is not only important but also essential for harnessing BF as a food pigment and a novel type of nutraceutical resource for future applications in the food industry. Consequently, this study aims to firstly investigate the in vitro gastrointestinal digestion model to assess the bioaccessibility of anthocyanins derived from BF. Secondly, it is imperative to determine the in vivo bioavailability of anthocyanins derived from BF, as well as their absorption kinetics and metabolic fate. Ultimately, an investigation into the antioxidant properties of BF extracts rich in anthocyanins should be conducted in vivo to elucidate their impact on the host’s enzymatic antioxidant defense.

## 2. Materials and Methods

### 2.1. Materials and Reagents

The butterfly flower was cultivated in Yunan, China, and air dried at room temperature after harvesting. The package of grounded powder of dried flower samples produced on 12 October 2023 with one year shelf life were purchased from Yunan, China and stored in a cool, ventilated, and dry place. The standard substances, including delphinidin 3-glucoside, cyanidin-3-glucoside and 4-Hydroxybenzoic acid were purchased from Yuanye Bio-Technology (Shanghai, China). Formic acid, methanol, acetonitrile, and other reagents were obtained from TEDIA (Fairfield, OH, USA). The activities of T-AOC, GSH, GSH-Px, SOD, and MDA were investigated by kits (Nanjing Jiancheng Bioengineering Co., Ltd., Nanjing, China).

### 2.2. Preparation of BF Extracts

Butterfly pea flower powder was extracted using a 70% (*v*/*v*) ethanol aqueous solution containing 0.5% citric acid (*v*/*v*). The extraction was performed three times using 1:10 (*w*/*v*) in a 100 W ultrasound bath for 15 min at each time. Subsequently, the mixture was centrifuged at 4000 rpm for 10 min. The combined supernatants were then evaporated under reduced pressure at 50 °C using a rotary vacuum evaporator(IKA, Staufen, Germany). The BF extract was freeze-dried and stored at −20 °C for further animal experiments.

### 2.3. In Vitro-Simulated Gastrointestinal Digestion

The in vitro digestion of BF extracts was performed based on the procedure described by Raffaella Colombo et al. [16], with slight modification. First, 600 mg butterfly pea flower extract was weighed and added to 5 mL HBSS buffer containing α-amylase (4000 U/g, final concentration 1.2 mg/mL). The mixture was cultured for 15 min at 200 rpm and 37 °C in a constant-temperature oscillating culture shaker. Subsequently, 6 N HCl was applied to adjust the pH of the mixture to 1.5. Meanwhile, the mixture was subjected to gastric digestion by porcine pepsin at 1.3 mg/mL and incubated for 1.5 h. For simulated intestinal digestion, KH2PO_4_ (1 mL, 0.5 M) was added to 4 mL PBS, 30 mg pancreatic enzyme, 6.66 mg CaCl_2_, and 30 mg bile salt were added to the abovementioned 5 mL solution, the pH was adjusted to 6.8 with NaHCO_3_ (1 M), and the temperature was kept at 37 °C for 2 h. The digestion was heated at 90 °C for 10 min to inactivate digestive enzymes, and finally centrifuged at 2000× *g* for 15 min (4 °C) to obtain the supernatant. The supernatant of pre-digestion, simulated gastric digestion, and simulated intestinal digestion was diluted to a certain multiple, divided into 2 mL centrifuge tubes, and stored in the refrigerator at −20 °C for later use. The experiment was divided into 3 groups as follows: NC: undigested group; GD: gastric digestion; ID: intestinal digestion.

### 2.4. Determination of Bioactive Content and Antioxidant Capacity

#### 2.4.1. Determination of Total Phenol Content

Folin–Ciocalteu method was applied to evaluate total phenolic content with 96-well plate according to our previous published procedure [17]. The 25 μL standard product (gallic acid) or sample was added into the 96-well plate, and then 125 μL 0.2 mol/L Folin reagent was added and incubated for 10min. After that, aliquoted 125 μL of saturated 10 g/100 mL Na_2_CO_3_ solution was added to continue incubation for 30 min at RT with slowly shaking on the oscillator. Finally, the absorbance was measured at 765 nm. The absorbance of 62.5, 31.25, 62.5, 125, 250, 500 µg/mL gallic acid was determined for standard curve. The content of total phenolic content was calculated based on standard curve.

#### 2.4.2. Determination of Total Flavonoid Content

The total flavonoid content was determined by sodium nitrite and aluminum trichloride. Briefly, 0.066 M NaNO_2_ and 25 μL standard (catechin) or sample were mixed at room temperature for 5 min, then 15 μL 0.75 M AlCl_3_ solution was added for 6 min, and finally 100 μL 0.5 M NaOH solution was added, which was slowly shaken at room temperature and reacted on an oscillator for 10 min. Absorbance was measured at 510 nm. The absorbance of 15.625, 31.25, 62.5, 125, 250, and 500 µg/mL catechin was determined under the same conditions, and the total flavonoid content of the sample was calculated based on the standard curve.

#### 2.4.3. Ferric Reducing Antioxidant Power (FRAP) Assay

The 10 μL FeSO_4_ series standard (0, 0.1, 0.5, 1, 2, 4, 6 mmol/L) or sample and 180 μL FRAP working liquid were added to the 96-well plate, and the absorption value was measured at 593 nm after incubation for 10 min in the dark. The antioxidant activity of the sample is expressed as the millimolar number of FeSO_4_ required to achieve the same absorbance.

#### 2.4.4. Determination of 1,1-Diphenyl-2-picrylhydrazyl (DPPH) Free Radical Scavenging Ability

The DPPH methanol solution of 100 μL 0.065 mM and 100 μL sample or blank solution were added into the 96-well plate, slowly shaken on the oscillator under the condition of dark light and reacted at room temperature for 30 min. The light absorption value was determined at 517 nm. With Trolox as the standard, the DPPH clearance capacity of the sample is expressed in milligrams of Trolox (mg TE/g DW) required to achieve the same elimination rate. The clearance rate is calculated as follows:

$$\mathrm{Elimination}\;\mathrm{ratio}/\%=(1-\frac{A_{i}-A_{j}}{A_{c}})\times 100\%$$

where *A_i_* is the absorbance of the mixture of sample solution and DPPH reagent, *A_j_* is the absorbance of sample solution and methanol, and *A_c_* is the absorbance of DPPH solution and sample solvent.

#### 2.4.5. Determination of 2,2′-Azino-bis(3-ethylbenzothiazoline-6-sulfonic Acid) (ABTS) Free Radical Scavenging Ability

Sample or standard solution (20 μL) and ABTS solution (200 μL) were added into the 96-well plate, respectively, and the reaction was slowly shaken on the oscillator at room temperature for 6min under the condition of avoiding light. The light absorption value was determined at 734 nm using Trolox as the standard. The ABTS clearance capacity of the sample is expressed as the number of milligrams of Trolox (mg TE/g DW) required to achieve the same elimination rate. The clearance rate is calculated as follows:

$$\mathrm{Elimination}\;\mathrm{ratio}/\%=(-\frac{A_{i}-A_{j}}{A_{0}})\times 100$$

where *A*_0_ is the absorbance of ABTS solution and sample solvent, *A_i_* is the absorbance of ABTS solution and sample solution, and *A_j_* is the absorbance of 80% methanol and sample solution.

### 2.5. Animal Experimental Design for Metabolic Study and Pharmacokinetic Experiments

A total of 24 male SD rats aged between 6 and 8 weeks (200–220 g) were acquired from the Laboratory Animal Center of Jiangxi University of Chinese Medicine (Nanchang, China). Prior to experimentation, the rats were acclimated for 7 days in an air-conditioned room maintained at 24 ± 1 °C with a 12 h light/dark cycle. During this period, they had free access to food and water. After adaptive feeding, rats were randomly divided into three groups (*n* = 8), including a negative control, serum collection, and urine collection groups. After being fasted for 12 h before the experiment, both serum and urine collection groups of mice were supplemented with 500 mg/kg BW of BF extracts. To establish baseline data, blood samples were collected from all rats before administering the BF extracts. Blood was collected from the orbits at 15 min, 30 min, 1 h, 1.5 h, 2 h, 4 h, 6 h, 8 h, and 10 h after drug administration. Urine was collected at 6, 12, and 24 h after administration. Pretreatment of serum and urine samples was according to our previous study [18]. The animal experiments were approved by the Animal Ethics Committee of Jiangxi University of Chinese Medicine (TEMPOR20230112).

#### 2.5.1. Identification and Quantification of Metabolites in Rats

The metabolic profile from blood and urine samples were conducted by UPLC-LTQ-Orbitrap-MS2 (Thermo Scientific, Bremen, Germany) according to previously published methods [6]. The samples were prepared by SPE, centrifuged at 4 °C and 2000 rpm, and stored at −80 °C. The samples were reconstituted with 100 uL of 80% methanol and 0.1% formic acid, and analyzed by UPLC-LTQ-Orbitrap-MS2 using an AC18 column (2.1 × 100 mm, 1.7 μm) to separate at 25 °C with a water/acetonitrile mobile phase with a 0.1% formic acid gradient. The mobile phase consisted of A (water containing 0.1% formic acid, *v*/*v*) and B (acetonitrile containing 0.1% formic acid, *v*/*v*), and the following binary program was used: 0–2 min, 5% B; 2–9 min, 5–21% B; 9–16 min, 21–29% B; 16–22 min, 29–55% B; 22–25 min, 55% B; 25–35 min, 55–100% B; 35–35.1 min, 100–5% B; 35.1–38 min, 5% B. The flow rate was 0.3 mL/min, and the injection volume was 2 μL. The LTQ-Orbitrap-MS2 was operated in negative mode with a scan range of 50–2000 *m*/*z*. A data-dependent scan with CID was performed for fragmentation. The collision energy was set at 45 eV. Data acquisition and processing were performed with Xcalibur v.2.0 software (Thermo Fisher, San Jose, CA, USA) and Qualbrowser.

The quantification of substances identified was performed using an AB TRIPLE QUAD 4500 HPLC-MS2 (AB SCIEX, Framingham, MA, USA) system equipped with a C18 column (2.1 × 100 mm, 1.7 μm particle size; ACQUITY) at room temperature (25 °C). The mobile phase consisted of A (water containing 0.2% formic acid, *v*/*v*) and B (acetonitrile containing 0.2% formic acid, *v*/*v*) and the following binary program was used: 0–3 min, 5% B; 3–4 min, 95% B; 4–4.1 min, 95–5% B; 4.1–6.0 min, 5% B. The flow rate was 0.3 mL/min, and the injection volume was 10 μL. For sensitive detection and quantification, the analysis employed electrospray ionization (ESI) followed by multiple reaction monitoring (MRM) to selectively monitor the fragmentation patterns of the target analytes. Data acquisition and processing were performed with Qualbrowser.

#### 2.5.2. Pharmacokinetic Analysis

Blood concentration/time data analysis was performed by non-compartment model analysis and processed using Peak View^®^1.6 (AB SCIEX), according to a previous publication [19]. All data are presented as mean ± standard deviation (SD). The maximum plasma concentration (Cmax), and the time to reach the maximum plasma concentration (tmax) was calculated from the observed values.

### 2.6. Animal Experimental Design for In Vivo Antioxidant Activities

A total of 32 SD male rats (200−220 g, 6−8 weeks of age) were randomly divided into four groups (*n* = 8) as follows:

NC: normal control (treated with saline);

PC: positive control (treated with D-galactose solution, 200 mg/kg BW);

BFL: low doses of BF extract (treated with 200 mg/kg BW BF extracts);

BFH: high doses of BF extract (treated with 500 mg/kg BW BF extracts).

The oxidative stress was induced using a D-galactose solution, following protocols from our previous publication [20]. This study was conducted with the approval of the Animal Ethics Committee of Jiangxi University of Chinese Medicine, adhering to ethical guidelines for animal research (protocol number: TEMPOR20230138). D-galactose solution (200 mg/kg BW) once a day for 20 days was used to induce oxidative damage. At the same time, rats in BF extracts treatment group were supplemented with 200 mg/kg BW and 500 mg/kg BW BF extracts by oral gavage. At the end of the experiment, the rats were euthanized using cervical dislocation. Plasma and liver samples were collected to analyze antioxidant markers.

#### In Vivo Antioxidant Activities

Plasma samples were centrifuged at 2500× *g*, and the supernatant was collected for analysis. Liver organ samples were cleaned, weighed, and homogenized in a 10 mM Tris-HCl buffer (pH 7.4). The homogenates were then centrifuged, and their supernatants were similarly collected. These supernatants were used to determine the activities of total antioxidant capacity (T-AOC), glutathione peroxidase (GSH-Px), superoxide dismutase (SOD), glutathione (GSH), and malondialdehyde (MDA) levels, following both the manufacturer’s instructions and the protocol described by Guo et al. [20]. All experiments were conducted at 4 °C. Enzyme activities in plasma and tissue were expressed as units per milliliter (U/mL) and units per milligram of protein (U/mg protein), respectively.

### 2.7. Statistical Analysis

Results are presented as mean values ± standard error of the mean (SEM). Data were analyzed using one-way ANOVA followed by Tukey’s multiple-comparison test or a one-tailed, unpaired Student’s *t*-test (GraphPad Prism version 9.0). When comparing the means of more than two groups, one-way ANOVA followed by Tukey’s multiple-comparison test was used. When comparing the means of two groups, a one-tailed, unpaired Student’s *t*-test was used. Differences were considered significant if the *p*-value was less than 0.05. Values without a common letter were significantly different at *p* < 0.05.

## 3. Results and Discussion

### 3.1. Stability and Antioxidant Activities of BF Extracts in a Simulated Gastrointestinal Tract Model

**Figure 1:**
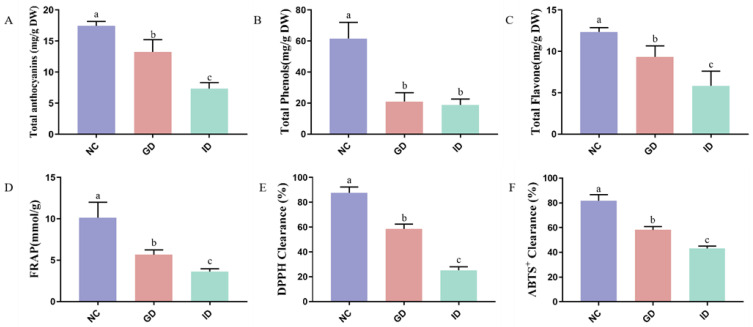
Study on the contents of phenolic substances and antioxidant activities in the extracts of butterfly bean flower. (**A**) Total anthocyanin content; (**B**) total phenol content; (**C**) total flavone content; (**D**) antioxidant capacity of FRAP; (**E**) DPPH clearance capacity; (**F**) ABTS clearance capability. The NC group simulated gastric fluid before digestion, the GD group simulated gastric fluid, and the ID group simulated intestinal fluid. Values without a common letter are significantly different at *p* < 0.05.

To investigate the bioaccessibility of BF extracts, the impact of the digestion process on the contents of the featured bioactives and associated bioactivities was evaluated in the present study via in vitro gastrointestinal digestion. In this study, the in vitro-simulated gastric and intestinal digestion results in a noticeable decrease in total anthocyanins (TAC), phenolic (TPC) and flavone (TFC) content in BF extracts and their mediated antioxidant activities, including FRAP, DPPH, and ABTS values (Figure 1A–F). Subsequently, around 75.89 ± 5.64% of TAC remained in BF extracts after gastric digestion, while 42.03 ± 2.74% TAC remained after intestinal digestion, respectively. Thus, a significant reduction in total anthocyanins was observed at the different stages of digestion process, specifically at the stage of intestinal digestion, which agreed with our previous finding [21]. This indicated that the digestion process has a strong impact on availability of anthocyanins derived from BF extracts.

Commonly, the anthocyanins are stable under the acidic conditions of the vacuoles of a plant cell. Several factors during digestion, like stomach acid, body temperature, and gut bacteria, can alter the structure of dietary anthocyanins [7]. During the digestive process, the significant anthocyanins degradation occurs during the intestinal digestion due to high content of digestive enzymes and basic condition [22]. Moreover, the BF was found to be rich in polyacylated anthocyanins, including a series type of ternatins A–D, as well as flavonoids such as kaempferol and quercetin glycoside derivatives [6]. According to a previous finding, a significantly higher degree of reduction was identified in nonacylated anthocyanins after intestinal digestion compared with acylated anthocyanins [23]. Moreover, a total loss of 39% of TAC and 37% of TPC were identified in purple tomato extracts after gastric digestion. The purple tomato was also found to have a high content of acylated anthocyanins. However, the different loss rate of TAC in the BF extract and purple tomato extract were revealed, suggesting that the structural attributes play a critical role in stability of natural anthocyanins derived from their food resources. Ternatins rich in BF are polyacylated derivatives of delphinidin 3,3′,5′-triglucoside [2]. The stability of anthocyanins enhanced by acylation depends on the acyl group and its structure, size, number, and attachment sites [24]. The stabilization of ternatins by hydroxycinnamic acids occurs through intramolecular or intermolecular co-pigmentation reactions, resulting in a reduction in polarity and steric hindrance to prevent nucleophilic attack [4]. The robust stability of anthocyanins derived from BF extracts enhances their antioxidant activities. Ultimately, as evidenced by the results, the variations in antioxidant activities of BF extracts during the in vitro-simulated digestion process align with their characteristic bioactive constituents. Our results suggested that the changes in pH and digestive enzymic activity as well as bile salts have a direct impact on the stability of BF extract-derived anthocyanins that leads to the alteration of its antioxidant activities.

### 3.2. The Pharmacokinetics of Key Anthocyanins and Their Metabolites in Rat

**Figure 2:**
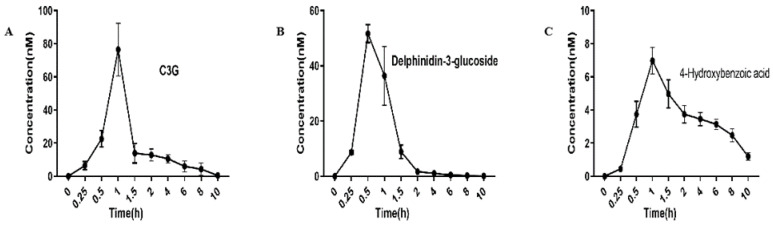
Serum pharmacokinetic profiles of (**A**) C3G, (**B**) D3G, and (**C**) 4-HBA in rats after the consumption of 500 mg/kg BW of BF extracts in eight rats. All data are mean ± SEM, *n* = 8.

The present study identified pharmacokinetic profiles of anthocyanins metabolites from rat plasma supplemented with BF extracts. Our previous research has confirmed the presence of twenty compounds, including flavonols (rutin, quercetin 3-O-rhamnodise, kaempferol 3-neohesperidoside) and anthocyanidins (pelargonidin-3-O-hexoside, delphinidin-3-(6″-*p*-coumaroyl)-rutinoside, ternatin D1, cyanidin-3-O-glucoside (C3G), and delphinidin-3-glucoside (D3G)) in BF extracts [6]. Among them, the intake form of C3G and D3G as well as their metabolite 4-hydroxybenzoic acid (HBA) have been detected in rat plasma after administration of BF extracts (Figure 2). However, the intact ternatin D1 was not detected in the present study. As shown in Table 1, the Cmax of C3G and D3G were assessed in plasma as 69.034 ± 8.05 nM and 51.65 ± 3.205 nM with tmax at 1 h and 0.5 h, respectively. The higher Cmax of C3G compared to D3G, and the almost twofold increase in area under the curve from 0 to 10 h (AUC_0–10_) for C3G, suggest a greater systemic circulation of C3G following administration of BF extracts. Nevertheless, ternatin D1, being polyacylated derivatives of glycosylated delphinidin, were not detected in rat blood samples, which is possibly due to its low bioavailability [25]. Despite the absence of detectable levels of intact ternatin D1 in rat serum, our study confirms the rapid absorption of D3G, consistent with a previous observation. A rapid peak of nasunin from eggplant (delphinidin-3-p-coumaroyl-rutinoside-5-glucoside) was identified in rat plasma at the 15 min postprandial time point, demonstrating comparable absorption kinetics to delphinidin-3-glucoside [8].

Moreover, since the circulated anthocyanin content is low in blood, the role of phenolic metabolites derived from anthocyanin in the health enhancement needs to be investigated rather than intact anthocyanin with low bioavailability. As detected in this study, the degradation product 4-HBA was shown at Cmax of 6.98 ± 0.805 nM when C3G reached a maximum serum concentration of 69.034 ± 8.05 nM of 4-HBA. However, no sufficient difference was observed between the tmax of C3G and 4-HBA. That is possibly due not only to the 4-HBA not being degraded from C3G but also involving D3G or other anthocyanins. A significant increase in 4-HBA levels has been observed in health subjects after consumption of organic products, indicating that 4-HBA is a key bioactive phenolic metabolite in the body [26].

### 3.3. Metabolic Fate of Key Anthocyanins Derived from BF in Rat Plasma and Urine

**Figure 3:**
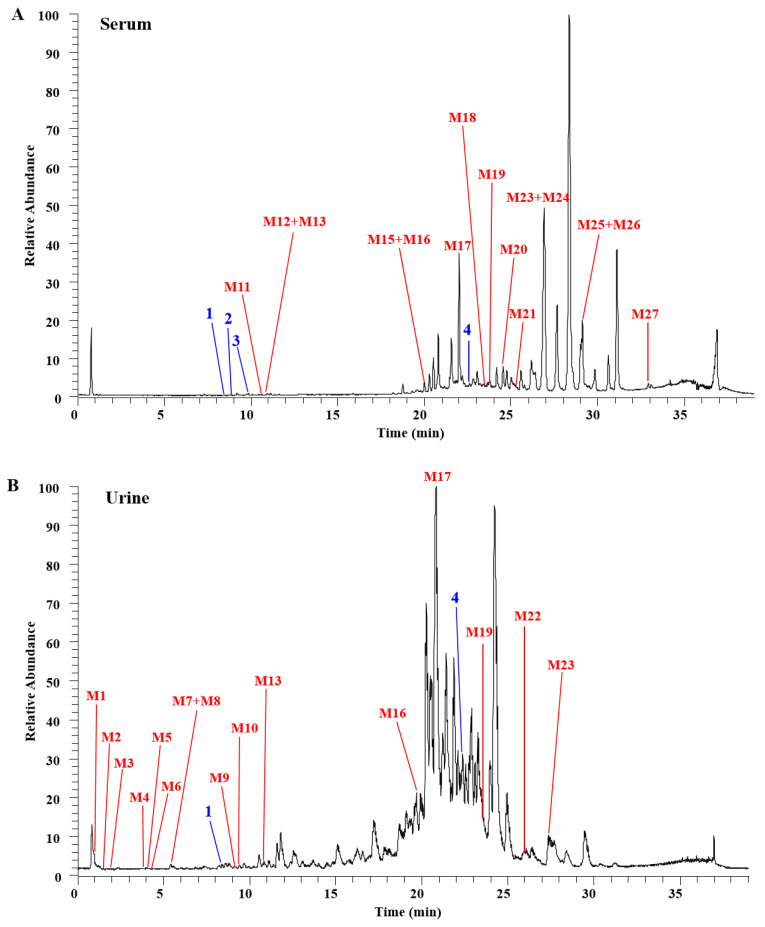

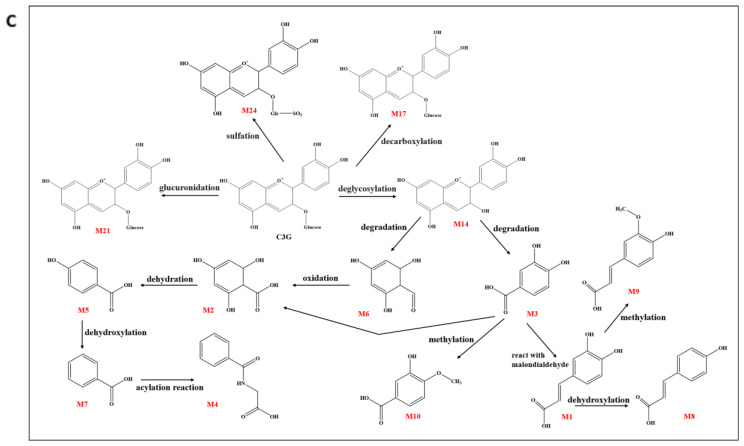
Total ion chromatograms (TIC) of (**A**) serum sample, (**B**) urine sample, and (**C**) characterization of the metabolites of C3G. Peaks marked with 1–4 are related to prototypes, and M1–M27 corresponded to the 27 metabolites of BF.

Limited knowledge exists regarding the postprandial metabolism of acylated anthocyanins derived from plant resources. Serum and urine samples were collected at various time points following gavage administration of BF extracts to investigate phase I and II metabolism. As shown in Figure 3 and Table 2, a total of four prototypes and twenty-seven metabolites were identified by comparing their retention time and fragmentation patterns to those of pure standards and existing literature. In this study, both D3G and C3G were detected in both serum and urine in the present study, while rutin and cyanidin-3-(*p*-coumaroyl) glucose were found exclusively in urine and serum, respectively. The presence of the prototype compound in serum suggests direct absorption through the gastrointestinal tract, whereas its excretion via urine may occur either directly or as a result of plasma metabolism. It is well established that flavonols and anthocyanidins are not only absorbed as their original forms, but also exert biological activity in the form of metabolites [27]. The presence of metabolites in serum and urine confirmed the in vivo metabolism of bioactive compounds from BF. These compounds underwent phase I transformations including dehydration, dehydroxylation, decarboxylation, and deglycosylation, followed by phase II conjugation reactions involving methylation, sulfation, and glucuronidation. The potential metabolic pathways of C3G, D3G, and rutin were described as follows.

#### 3.3.1. Metabolic Profiling of C3G

The precursor ion of M14 was observed at *m*/*z* = 285.11, which indicated a mass reduction in 162 Da (corresponding to Glc) compared to C3G. It exhibited identical fragment ions to those of C3G and was identified as cyanidin (Cy), the deglycosylation metabolite of C3G. M3 and M6 shared the same precursor ion at *m*/*z* = 153.02, suggesting their formation as degradation products of Cy. Through comparison of MS/MS fragments, M3 and M6 were characterized as protocatechuic acid (PCA, B-ring moiety of Cy) and phloroglucinaldehyde (PGA, A-ring moiety of Cy), respectively. Additionally, M2 displayed a precursor ion at *m*/*z* = 169.01, indicating a mass reduction by 16 Da (representing oxygen) compared to PGA; this compound was identified as 2,4,6-trihydroxybenzoic acid (THBA), an oxidation product derived from PGA. The precursor ion of M5 was observed at *m*/*z* = 137.02, which exhibited a mass decrease of 32 Da (2OH) compared to THBA, indicating the formation of a dehydration product identified as 4-hydroxybenzoic acid (4-HBA). The precursor ion of M7 appeared at *m*/*z* = 120.05, showing a mass reduction of 17 Da (OH) relative to 4-HBA, suggesting the generation of a dehydroxylation product known as benzoic acid (BA). M4 displayed a precursor ion at *m*/*z* = 178.05, exhibiting an increase in mass by 58 Da compared to BA, implying an acylation reaction with glycine leading to the formation of hippuric acid (HA). Therefore, it can be inferred that M2, M4, M5, and M7 are putative phase I and phase II metabolites derived from PGA based on previous studies [9,28]. While PCA and PGA share the same precursor ion, 4-HBA, BA, and HA may also serve as metabolites of PCA. By comparing with the existing literature, it has been observed that PCA can undergo alternative metabolic pathways such as methylation and react with malondialdehyde [29]. This is attributed to the substitution of methyl groups with hydroxy groups in phase I metabolism followed by methylation through the addition of methyl groups to hydroxy groups in phase II mediated by cytochrome P450 [30,31]. M10 showed precursor ion at *m*/*z* = 167.02, which were 14 Da (CH2) more than PCA and supposed to be the methylation product of PCA (vanillic acid, VA). M1 exhibits a precursor ion at *m*/*z* = 179.06, which is 26 Da higher than that of PCA, indicating a reaction with malondialdehyde and subsequent production of caffeic acid (CA).

The precursor ion M9, detected at *m*/*z* = 193.05 with a mass difference of 14 Da (CH2) compared to CA, was postulated as the methylated product of CA (ferulic acid, FA). On the other hand, M8, observed as a precursor ion at *m*/*z* = 162.06 and exhibiting a mass reduction in 17 Da (OH) relative to CA, was proposed as the dehydroxylated derivative of CA (p-coumaric acid, p-CA). Consequently, M1, M2, M4, M5, M7, M8, M9, and M10 were postulated as potential phase I and phase II metabolites of PCA. Furthermore, several sulfation (M22, M24) and glucuronidation (M11, M12, M13, M19, and M21) metabolites were provisionally identified. The detailed metabolic pathway of C3G was depicted in Figure 3C. Notably, these metabolites have been reported to exhibit potent bioactivities, such as antioxidation.

#### 3.3.2. Characterization of the Metabolites of D3G

Delphinidin is composed of ternatins, which are recognized as the characteristic acyl anthocyanins in BF extract [3]. Both phase I and II metabolites of the compound were detected in both rat plasma and urine samples. M16 was identified with a precursor ion at *m*/*z* = 302.04, which is 162 Da lower than D3G, indicating deglycosylation of D3G (delphinidin). In addition, a precursor ion at *m*/*z* = 640.28 was identified in M26, which was 176 Da more than D3G. This observation suggests the presence of a glucuronidation and methylation product derived from D3G in M26 [32]. Lastly, M27 displayed a precursor ion at *m*/*z* = 655.42 with an additional mass of 15 Da relative to M26, indicating the formation of a glucuronidation and methylation product originating from D3G. Consistent with previous findings, intact acylated anthocyanins and their glucuronide derivatives were not detected in rat serum and urine samples, indicating that the acyl groups of anthocyanins derived from BF extracts are deacylated prior to undergoing phase I and II metabolism in the liver [8,22]. Our current research demonstrates that nonacylated D3G, originating from BF, undergoes conjugation to generate both glucuronide and methylated metabolites in the serum and urine samples of rats.

#### 3.3.3. Characterization of the Metabolites of Rutin

Analysis of serum and urine samples revealed the presence of M23 and M25, with precursor ions at *m*/*z* = 564.33 and 566.33, respectively, indicating that they are metabolites of rutin after loss of HCOOH and COO groups, corresponding to a mass reduction of 46 Da and 44 Da, respectively [33]. Furthermore, Figure 3 and Table 2 provide detailed information on other phenolic metabolites identified in rat serum and urine following intake of BF extracts. Accordingly, BF extracts contained rutin (quercetin-3-O-rutinoside), which is a primary flavonoid and recognized as a co-pigment for stabilizing the natural blue color of anthocyanins. These in vivo-synthesized phenolic metabolites and derivatives may possess significant health-promoting properties warranting further investigation.

### 3.4. In Vivo Antioxidant Activities of BFs

Consuming bioactive compounds from food sources can facilitate their release during digestion. As natural pigments, anthocyanins and flavonoids from plant foods are subject to extensive biotransformation in vivo, which leads to the creation of a variety of bioactive metabolites [22,34]. BF has a high content of polymer acyl anthocyanins that improve the stability and bioavailability of their parent anthocyanins [7]. This suggests that these compounds are effectively absorbed and utilized by the human body. In vivo, the conjugated forms of anthocyanin metabolites are more prevalent than their native dietary counterparts, leading to substantial health benefits for the host. These benefits include the scavenging of reactive oxygen species (ROS) and the enhancement of antioxidant enzyme functions [31,35]. Hence, the impact of supplemented BF extracts on antioxidant enzyme (i.e., SOD, GSH-Px activity, and CAT) activities were evaluated using D-galactose-induced rat model in the present study. Furthermore, D-galactose, known as a monosaccharide, has been extensively utilized to induce oxidative stress in rats and thus contributes to the accumulation of age-related deficits [36,37]. Anthocyanins have been demonstrated to ameliorate D-galactose-induced oxidative stress and neuroinflammatory damage in a rat model [38]. As the in vivo biological activity of polyphenols serves as a crucial indicator of their bioavailability, an oxidative stress rat model was employed to investigate the antioxidant potential of BF-derived anthocyanins.

**Figure 4:**
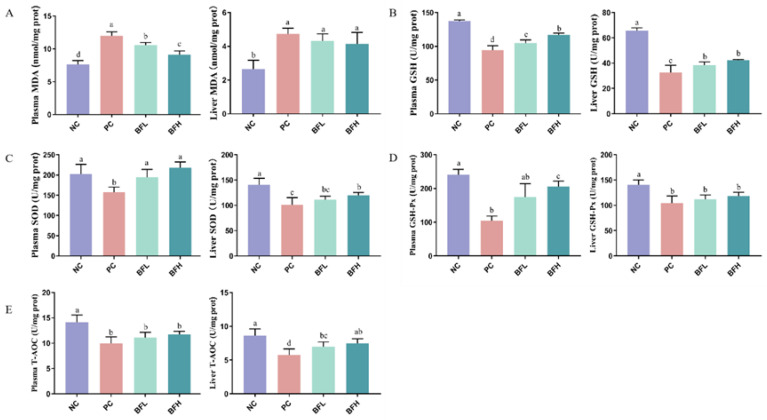
Effects of BF extract on antioxidant activity in rat blood and liver. (**A**) MDA, (**B**) GSH, (**C**) SOD, (**D**) GSH-Px, (**E**) T-AOC. Data are presented as mean ± SD (*n* = 8); values not sharing a common superscript letter denote significant difference (*p* < 0.05). NC: negative control group; PC: positive control group; BFL: low-dosage group; BFH: high-dosage group.

Based on our findings, a statistically significant difference (*p* < 0.05) was observed between the NC and PC groups (Figure 4). This suggests that D-galactose treatment induced oxidative stress in the rats. As shown in Figure 4A, either the low-dose (BFL) or high-dose (BFH) extracts significantly decreased the levels of MDA in both the rat plasma and liver tissues compared to the PC group. A noticeable elevation in MDA levels in the plasma indicated a systemic lipid oxidation developed in D-galactose-induced rats. This is consistent with previous finding in which a higher systemic oxidant stress was identified in the aging group [39]. As shown in Figure 4A, administration of BF extracts significantly suppressed the plasmic MDA level compared to that of PC group. Similarly, the intake of black chokeberry extract with high anthocyanin content was shown to effectively reduce the MDA level in plasma and liver from D-galactose-induced mice [40]. Moreover, the higher doses of BF extract showed a stronger inhibitory effect on the production of lipid peroxidation. The stronger antioxidant activity of acylated anthocyanins compared to their corresponding nonacylated compounds has been observed [41]. The current study showed that supplementation of anthocyanin-rich foods, including berries, vegetables, and cereals, can significantly prevent development of cardiovascular disease (CVD) by reducing lipid peroxidation while restoring plasma antioxidant capacity [42]. Furthermore, supplementation of BF extracts was shown to significantly increased activity of SOD and GSH-Px enzymes in both their blood plasma and liver samples (Figure 4C,D). Interestingly, higher extract doses resulted in proportionally stronger enzyme activity. However, only high doses of the extract significantly increased total antioxidant capacity (T-AOC) across all tissues examined (Figure 4E). Since SOD and GSH-Px are crucial components of the body’s natural defense system, their increased activity by the BF extract, particularly at higher doses, suggests a beneficial effect. This enhancement likely helps the rats combat oxidative stress and minimize cell damage. A previous finding demonstrated that both purple potatoes and carrots are rich in acyl anthocyanins that exert a strong effect on restoring the intrinsic antioxidant defenses by elevating SOD and GR/GPx activities against reactive oxidant species-induced cell damage [13]. Moreover, the administration of BF extract significantly increased the GSH content in rat plasma and the liver (Figure 4B). The results indicate that BF extract can shield cells from oxidative damage by lowering MDA, a marker of lipid peroxidation, and elevating GSH, a key antioxidant. Observations from a current study showed that the administration of protein-conjugated anthocyanin improved the GSH content in liver and decrease oxidative stress and associated liver damage in a diabetic mouse model [43]. The aging process is associated with a prolong oxidative stress that alerts the normal physiological status and increases the risk of developing chronic disease [44,45]. The daily intake of dietary sources of natural antioxidants such as polyphenols, being widely contained in plant-based foods as natural pigments, can effectively improve antioxidant defense mechanisms that prevent oxidative stress [11,46]. Our finding also suggests that acyl anthocyanins may potentially contribute to the reactivation of antioxidant enzymes, thereby enhancing the antioxidant protective activity derived from BF in an animal model.

## 4. Conclusions

Butterfly pea flower (BF) has gained attention for its rich content of anthocyanins and associated antioxidant properties. Thus, this study explored how digestion and metabolism affect BF extracts and their subsequent antioxidant activity within the body. As revealed in the present study, around three quarters of total anthocyanins survived gastric digestion, and half remained after intestinal digestion. The trend of changes in antioxidant activities were in line with the changes in total anthocyanins as observed from in vitro digestion experiment. In vitro digestion experiments revealed a decrease in phenolic content and antioxidant activity of BF extracts. This suggests that digestion may affect the bioaccessibility of BF-derived anthocyanins due to degradation. Furthermore, the postprandial metabolism was analyzed by UPLC-LTQ-Orbitrap-MS^2^. The pharmacokinetics of key anthocyanins including C3G and D3G and their metabolite 4-HBA were assessed and found to have a higher C_max_ and AUC_0-10_ of C3G compared to D3G, suggesting a higher bioavailability of C3G after the administration of BF extracts. The serum profile was subsequently investigated and identified 32 metabolites in rat urine and plasma samples after BF extract intake. These metabolites likely originated from BF’s anthocyanins. The metabolic fate of key metabolites derived from BF was revealed in the present study, including C3G, D3G, and quercetin. These metabolites demonstrated strong antioxidant properties. In a D-galactose-induced rat model, they even enhanced the activity of antioxidant enzymes SOD, GSH-Px, GSH, T-AOC, and MDA in both the blood plasma and liver. Overall, this research highlights the potential benefits of butterfly-derived anthocyanins through exploring the impact of digestion process on their bioaccessibility, postprandial metabolic fat of BF-derived anthocyanins and their antioxidant activities. This knowledge is crucial for understanding the true effectiveness of BF that warrants their future application in functional foods. To fully elucidate the mechanisms underlying these observations, further research is warranted. This will bridge the knowledge gap in comprehending the impact of various structural anthocyanins on health outcomes.

## Figures and Tables

**Table 1:** Serum pharmacokinetic profiles of C3G, D3G, and its degradation product 4-HBA in rats after the consumption of 500 mg/kg BW of BF extracts.

Metabolite	Cmax (nM)	tmax (h)	t1/2 (h)	AUC0-10 (nmol h L^−1^)
Delphinidin 3-glucoside	51.65 ± 3.201	0.5	1.639 ± 0.271	54.654 ± 9.089
Cyanidin-3-glucoside	69.034 ± 8.051	1	1.214 ± 0.38	131.314 ± 12.185
4-Hydroxybenzoic acid	6.98 ± 0.805	1	6.483 ± 5.165	31.279 ± 1.908

**Table 2:** Characterization of rat metabolites of BF extracts by UPLC-LTQ-Qrbitrap-MS^2.^

Peak #	RT (min)	[M-H]^−^ (*m*/*z*)	Tentative Identification	Fragments (MS^2^)	Location	Original Compounds
Original compounds						
1	8.40	464.30	delphinidin 3-glucoside	403.26, 367.26, 301.17, 196.00	S, U	-
2	8.96	610.42	rutin	564.39, 474.15, 302.06	S	-
3	9.81	593.15	cyanidin-3-(p-coumaroyl) glucose	447.19, 284.98	S	-
4	22.61	448.31	cyanidin-3-*O*-glucoside (C3G)	380.23, 285.11, 134.16	S, U	-
Metabolites						
M1	0.93	179.06	caffeic acid (CA)	-	U	C3G
M2	1.44	169.01	2,4,6-trihydroxybenzoic acid (THBA)	125.17	U	C3G
M3	1.70	153.02	protocatechuic acid (PCA)	81.17	U	C3G
M4	3.97	178.05	hippuric acid (HA)	135.06, 80.16	U	C3G
M5	4.00	137.02	4-hydroxybenzoic acid (4-HBA)	109.19, 108.28, 81.26	U	C3G
M6	4.29	153.02	phloroglucinaldehyde (PGA)	81.17, 108.56	U	C3G
M7	5.40	120.05	benzoic acid (BA)	93.11, 79.00, 64.91	U	C3G
M8	5.42	162.06	*p*-coumaric acid (*p*-CA)	144.20, 119.27, 74.22	U	C3G
M9	9.04	193.05	ferulic acid (FA)	178.01, 149.08, 133.99	U	C3G
M10	9.31	167.02	vanillic acid (VA)	-	U	C3G
M11	10.56	329.00	PCA-3-glucuronide	153.02	S	C3G
M12	10.93	343.08	VA-4-glucuronide	175.24, 169.19, 152.24, 113.25	S	C3G
M13	10.98	329.00	PCA-4-glucuronide	153.02	S, U	C3G
M14	11.86	285.11	cyanidin (Cy)	267.15, 223.19, 137.11, 81.30	U	C3G
M15	19.99	1746.38	dehydration	1708.66, 1534.82, 1406.51	S	ternatin D1
M16	20.00	302.04	deglycosylation	285.21, 247.06, 165.22	S, U	delphinidin 3-glucoside
M17	21.98	407.28	decarboxylation	386.30, 290.26	S, U	C3G
M18	23.54	431.19	deglycosylation	-	S	cyanidin-3-(*p*-coumaroyl) glucose
M19	23.98	315.00	benzoic acid-4-glucuronide	175.18	S, U	C3G
M20	24.53	446.23	dehydrogenation	427.14, 378.24	S	quercetin 3-O-rhamnoside
M21	25.37	624.31	glucuronidation	562.27, 494.47	S	C3G
M22	25.90	233.16	PCA-3-sulfate	189.04, 144.02, 83.17	U	C3G
M23	26.90	564.33	loss of HCOOH	428.03, 255.27	S, U	rutin
M24	26.94	528.31	sulfation	468.33, 303.21, 259.25	S	C3G
M25	29.10	566.33	loss of COO	506.33	S	rutin
M26	29.13	640.28	glucuronidation	621.17, 508.44, 437.40, 300.00	S	delphinidin 3-glucoside
M27	32.89	655.42	glucuronidation, methylation	610.29, 520.89, 348.24	S	delphinidin 3-glucoside

S: serum; U: urine.

## Data Availability

The original contributions presented in the study are included in the article, further inquiries can be directed to the corresponding author.
